# Precipitation and nitrogen addition enhance biomass allocation to aboveground in an alpine steppe

**DOI:** 10.1002/ece3.5706

**Published:** 2019-10-04

**Authors:** Changbin Li, Zhi Zheng, Yunfeng Peng, Xiuqing Nie, Lucun Yang, Yuanming Xiao, Guoying Zhou

**Affiliations:** ^1^ Key Laboratory of Tibetan Medicine Research Northwest Institute of Plateau Biology Chinese Academy of Science Xining China; ^2^ Qinghai Key Laboratory of Qing‐Tibet Biological Resources Xining China; ^3^ University of Chinese Academy of Science Beijing China; ^4^ State Key Laboratory of Vegetation and Environmental Change Institute of Botany Chinese Academy of Sciences Beijing China

**Keywords:** aboveground biomass, belowground biomass, nitrogen addition, precipitation changes, Tibetan alpine steppe

## Abstract

There are two important allocation hypotheses in plant biomass allocation: allometric and isometric. We tested these two hypotheses in an alpine steppe using plant biomass allocation under nitrogen (N) addition and precipitation (Precip) changes at a community level. An in situ field manipulation experiment was conducted to examine the two hypotheses and the responses of the biomass to N addition (10 g N m^−2^ y^−1^) and altered Precip (±50% precipitation) in an alpine steppe on the Qinghai–Tibetan Plateau from 2013 to 2016. We found that the plant community biomass differed in its response to N addition and reduced Precip such that N addition significantly increased aboveground biomass (AGB), while reduced Precip significantly decreased AGB from 2014 to 2016. Moreover, reduced Precip enhanced deep soil belowground biomass (BGB). In the natural alpine steppe, the allocation between AGB and BGB was consistent with the isometric hypotheses. In contrast, N addition or altered Precip enhanced biomass allocation to aboveground, thus leading to allometric growth. More importantly, reduced Precip enhanced biomass allocation into deep soil. Our study provides insight into the responses of alpine steppes to global climate change by linking AGB and BGB allocation.

## Graphical abstract

In summary, we demonstrated that altered precipitation and N addition led to changes in the relationship between aboveground and belowground biomass allocation. Moreover, we demonstrated that reduced precipitation had little impacts on aboveground and belowground biomass allocation, while it enhanced more biomass into deep soil. Therefore, our finding provides a novel insight into N and precipitation‐induced changes in the aboveground and belowground biomass allocation in the alpine steppe.

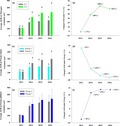

## 1 INTRODUCTION

Human activity‐induced climate change, especially in part due to shifts in precipitation patterns, and enhanced emission of biological reactive nitrogen (N) to the atmosphere have had profound impacts on the global water and N cycle (Basto et al., 2018; Stevens, 2019; Yu et al., 2019). As water and N are the most important factors that determine the growth and survival of plant and limit the production of grassland ecosystems (Bobbink et al., 2010; Greaver et al., 2016), the altered Precip and enhanced N deposition affect grassland ecosystem functions and services that are essential for well‐being of humanity (Basto et al., 2018; Liu et al., 2018). Despite numerous reports on the effects of altered Precip and enhanced N deposition grassland ecosystems (Liu et al., 2018; Ma et al., 2019; Tian et al., 2016; Yang et al., 2011), less is known with respect to how altered Precip, enhanced N deposition, and their interaction in the alpine steppe will influence aboveground and belowground biomass allocation, especially at different soil layer (Liu et al., 2018).

Aboveground and belowground biomass allocation reflected evolutionarily derived strategies for resource acquisition and adaptation to their environment (Lambers, Chapin, & Pons, 2008). Theoretically, plant should allocate more carbon to organs that acquire essential and limiting resources (Johnson, Rowland, Corkidi, & Allen, 2008). For example, as plant growth is limited by soil resources such as nutrient and water, plant species will allocate more carbon to root systems to acquire limiting soil resource (Johnson et al., 2008). In contrast, in light‐limited ecosystem, plant species will allocate more carbon to leaves to intercept light and fix CO_2_ for plant growth (Hautier, Niklaus, & Hector, 2009). This leads to allometric allocations that variations in environmental conditions affect aboveground and belowground biomass allocation (Bloom, Chapin, & Mooney, 1985; Chapin, Bloom, Field, & Waring, 1987). By contrast, plants rely on their homeostasis to cope with the environment, thus leading to an isometric allocation that aboveground biomass (AGB) scales with belowground biomass (BGB) in an isometric manner (Enquist & Niklas, 2002; Niklas, 2004). Previous studies found that, in community level of Tibetan Plateau grassland ecosystems, AGB and BGB allocation fitted the isometric hypothesis (McCarthy & Enquist, 2007; Yang, Fang, Ji, & Han, 2009), while on an individual level, the allocation fitted the allometric relationship (Wang, Niu, Yang, & Zhou, 2010), indicating that AGB and BGB allocation has strong scale dependence in grassland ecosystems of Tibetan Plateau grassland ecosystems. Plant growth in the alpine steppe is colimited by N and water (Hooper & Johnson, 1999). Thus, altered Precip and enhanced N deposition should have impacts on AGB and BGB allocation. However, we less known how altered Precip, enhanced N deposition, and their interaction had impacts on AGB and BGB allocation relationship.

Alpine ecosystem is believed among the most sensitive ecosystems to global changes (Chen et al., 2013). Alpine steppe is an integral part of the Qinghai–Tibetan Plateau. Human activities have led to dramatic changes in Precip and atmospheric deposition (Galloway et al., 2008; Peñuelas et al., 2013; Reay, Dentener, Smith, Grace, & Feely, 2008). For example, during the period 1990–2003, atmospheric N deposition has increased substantially from 8.7 to 13.8 kg N ha^−1^ year^−1^ in the region (Lü & Tian, 2007). Our precious studies have shown that altered precipitation and enhanced N deposition had a marked influence on aboveground community biomass (Li et al., 2018). However, we less known their responses of community BGB and allocation between AGB and BGB to altered Precip, enhanced N deposition, and their interaction in the alpine steppe. To address these issues, we investigated the biomass allocation and tested the allometric and isometric allocation hypotheses at the community level of alpine steppe under enhanced N deposition and altered precipitation patterns.

## 2 MATERIALS AND METHODS

### 2.1 Study site

The field experiment was conducted on the Sanjiaocheng Sheep Breeding Farm (37°18′N, 100°15′E, 3,286 m a.s.l), in Qinghai Province, China. The experiment site was located in an alpine steppe with mean annual temperature of 0.08°C and precipitation of 387 mm which occurs predominantly in the growing season (June to August). The soil belongs to chestnut soil. Vegetation is an alpine steppe community mainly dominated by *Stipa purpurea*, and accompanied by *Poa crymophila* and *Artemisia scoparia* (Peng & Yang, 2016).

### 2.2 Experimental design

The experiment was established in 2013 following exclusion of livestock grazing by fencing. The manipulative experiments include six treatments (N_1_P_1_: ambient N addition with reduced precipitation 50%; N_1_P_2_: ambient N addition with ambient precipitation; N_1_P_3_: ambient N addition with enhanced precipitation 50%; N_2_P_1_: N addition with reduced precipitation; N_2_P_2_: N addition with ambient precipitation; and N_2_P_3_: N addition with enhanced precipitation). Each treatment had five replicates, and 30 3.3 m × 2.7 m plots separated by 2‐m‐wide buffer strips were established in a 5 × 6 block design. The precipitation treatment was controlled by sunlight‐pervious concave polyvinyl chloride (PVC) boards (1 mm thick) at the 15° angle above each plot. The reduced precipitation treatment was controlled by the nonslotted channels, and 50% of the intercepted rainwater was collected and stored. The enhanced treatment was provided by slotted channels that sprinkled the collected water from the reduced plots immediately after the rain, resulting in a 50% increase relative to ambient precipitation. The ambient precipitation treatment plots were also installed with slotted channels. To avoid surface runoff, metal plates were inserted to a soil depth of 20 cm with 10 cm remaining above the surface around each plot. Moreover, to simulate ambient N deposition, NH_4_NO_3_ (10 g N m^−2^ year^−1^, N > 99%) was used in N addition plots. The fertilizers were mixed with water in 1‐L water by sprinkling evenly using a sprayer to each plot. Ambient N addition plots received equal dose of water. This N deposition rate is higher than the current natural N deposition rate (ranging from 0.87 to 1.38 g N ha^−1^ year^−1^) in this region, but atmospheric N deposition rates as high as 5.46 g m^−2^ year^−1^ in China (Xu et al., 2018). A recent study in the same region demonstrated that N addition rate of 8 g m^−2^ year^−1^ led to N saturation in soil (Peng & Yang, 2016).

### 2.3 Field sampling

Aboveground biomass was sampled in mid‐August annually by clipping all plants within three 0.25 m × 0.25 m quadrates that were randomly placed within each plot and not overlapped spatially among years. Briefly, shoots were cut at the soil surface and oven‐dried at 65°C for 72 hr before they were weighed (Li et al., 2018). Thereafter, to assess the effect of altered precipitation, N addition, and their interaction on over all belowground biomass and aboveground biomass at different soil layer, we collected belowground biomass using three 8‐cm‐diameter soil cores from each plot in mid‐August 2016. The soil cores were further divided into three soil layers: 0–10, 10–20, and 20–30 cm. Three soil cores from the same layers in each plot were mixed and placed into root bags with a mesh size of 0.5 mm and immersed in water for 24 hr. These soil cores were flushed with running water. These root samples were picked up by manual dissection as described by Zheng, Bai, and Zhang (2019). Then, they were oven‐dried at 65°C for 48 hr weighted. Total belowground biomass was determined by pooling over three layers.

### 2.4 Statistical analysis

To investigate N and precipitation‐induced changes of aboveground net primary productivity, we used date from 2013 to 2016 to assess the effect of N addition, precipitation change, and their interaction on aboveground biomass. Prior to all statistical analyses, we tested the heterogeneity of variances, and original data were normalized using log‐transformation or standardization prior to statistical analysis when necessary. Firstly, an univariate analysis of variance (UNIANOVA) was used to assess treatment effects on aboveground, in which N addition, precipitation change, and year were the categorical variables, whereas aboveground biomass was the continuous variable. For total belowground biomass, belowground biomass from different soil depth and the ratio of root to shoot (R/S), we used the data from 2016. Moreover, unpaired tests were also used to evaluate the effects of N addition, precipitation on aboveground biomass at the same year, total belowground biomass, R/S, and belowground biomass from the same soil depth. The statistical analyses above were performed in SPSS 16.0.

To examine the two allocation hypotheses allometric allocations and isometric allocation, reduced major axis (RMA) analyses were used to evaluate the relationship between log‐transformed aboveground biomass and belowground biomass (Cheng & Niklas, 2006; Niklas, 2004).The slope (*α*) and *y*‐intercept (log *β*) of the log–log linear functions were determined using the software package SMATR (Standardized Major Axis Tests and Routines; Falster, 2003; Warton, Duursma, Falster, & Taskinen, 2012) in the R Version 3.51 (R Development Core Team, 2018).

## 3 RESULTS

### 3.1 Effects of altered precipitation, N addition, and their interaction on aboveground biomass, belowground biomass, and aboveground–belowground biomass allocation

**Figure 1:**
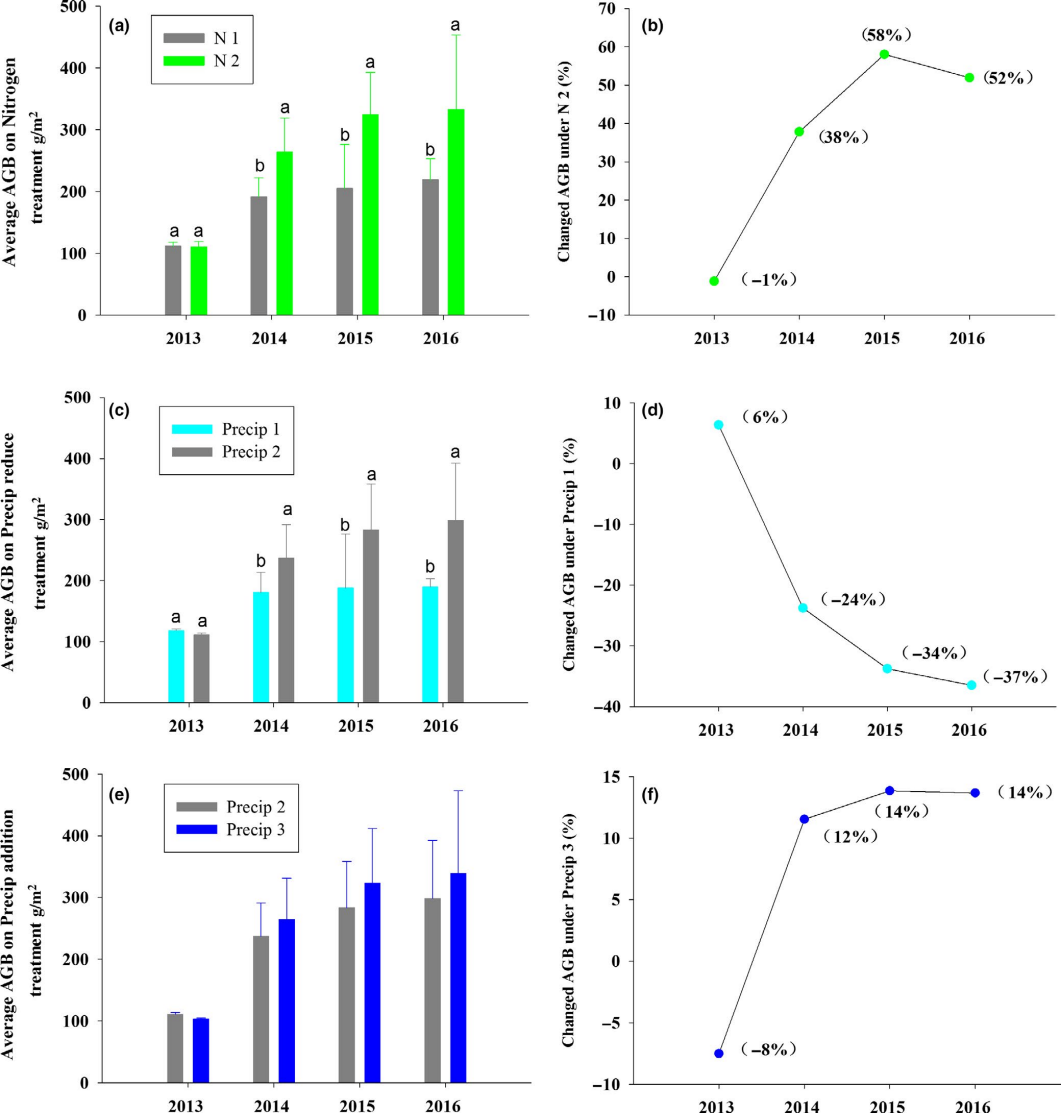
Effects of precipitation changes and N addition on aboveground biomass (AGB) (a, c, e) and changes (%) in aboveground biomass (b, d, f) from 2013 to 2016. N1 indicates ambient N, nitrogen addition (N2), 50% precipitation reduction (Precip 1), ambient precipitation (Precip 2), 50% precipitation addition (Precip 3), error bars indicate the standard errors

Aboveground plant biomass differed in response to N addition and altered Precip (Figure 1a–f; Table 1). For example, N addition significantly enhanced aboveground biomass by 38%, 58%, and 60% in 2014, 2015, and 2016, respectively (*p* < .05; Figure 1a,b). In contrast, reduced Precip led to a significant decrease in aboveground biomass by 24%, 34%, and 37% in 2014, 2015, and 2016, respectively (Figure 1c,d), while enhanced Precip had no effect on aboveground biomass (Figure 1e,f).

**Table 1:** Summary of univariate analysis of variance (UNIANOVA) of nitrogen addition and precipitation change on aboveground biomass from 2013 to 2016

Difference source	*df*	Mean square	*F*‐value	*p*‐value
Year (Y)	3	180,204.48	44.15	<.001
Nitrogen (N)	1	161,262.94	39.51	<.001
Precipitation (Precip)	2	47,129.98	11.55	<.001
Y × N	3	21,391.31	5.24	.002
Y × Precip	6	12,050.97	2.95	.011
N × Precip	2	1,965.08	0.48	.619
Y × N × Precip	6	2,590.45	0.64	.702

**Figure 2:**
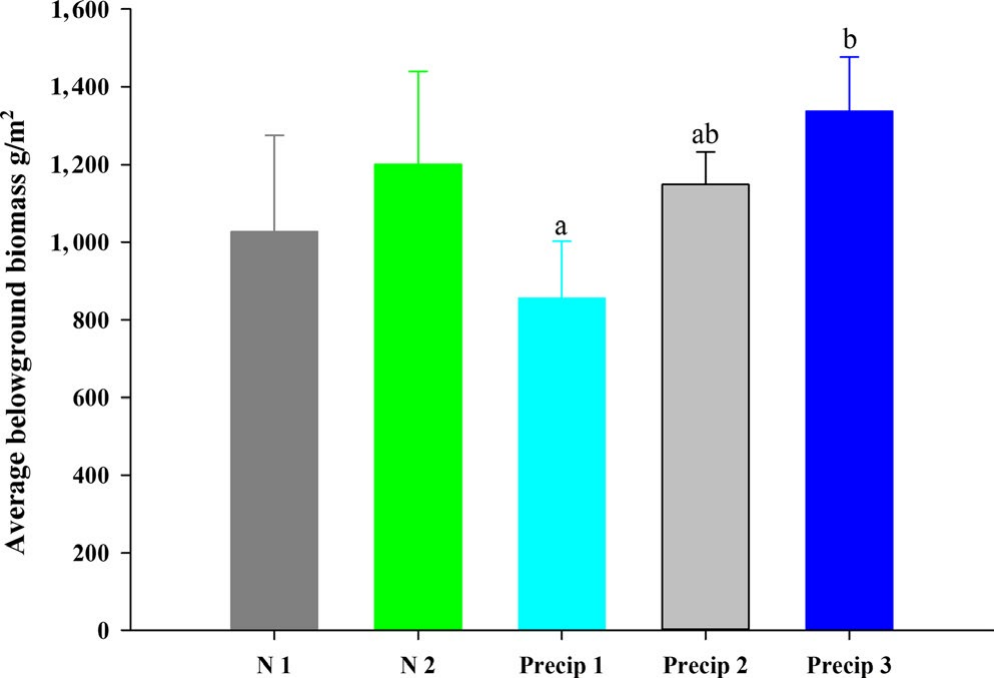
Effects of precipitation changes and N addition on belowground biomass (BGB) in 2016. N1 indicates ambient N, nitrogen addition (N2), 50% precipitation reduction (Precip 1), nature precipitation (Precip 2), 50% precipitation addition (Precip 3), error bars indicate the standard errors

In contrast to aboveground biomass responses, N addition had little impacts on overall belowground biomass (*p* = .20; Figure 2). Moreover, a differential response of belowground biomass to enhanced Precip and reduced Precip was observed after consecutive altered Precip for three years, such that enhanced Precip significantly increased belowground biomass by 16%, while reduced Precip led to a marked decrease in belowground biomass by 34% (*p* = .02; Figure 2; Table 2).

**Table 2:** Summary of univariate analysis of variance (UNIANOVA) of nitrogen addition and precipitation change on belowground biomass (BGB) in 2016

Difference source	*df*	Mean square	*F*‐value	*p*‐value
Nitrogen (N)	1	228,770.83	1.76	.20
Precip	2	588,523.30	4.52	.02
N × Precip	2	6,003.94	0.05	.10

**Figure 3:**
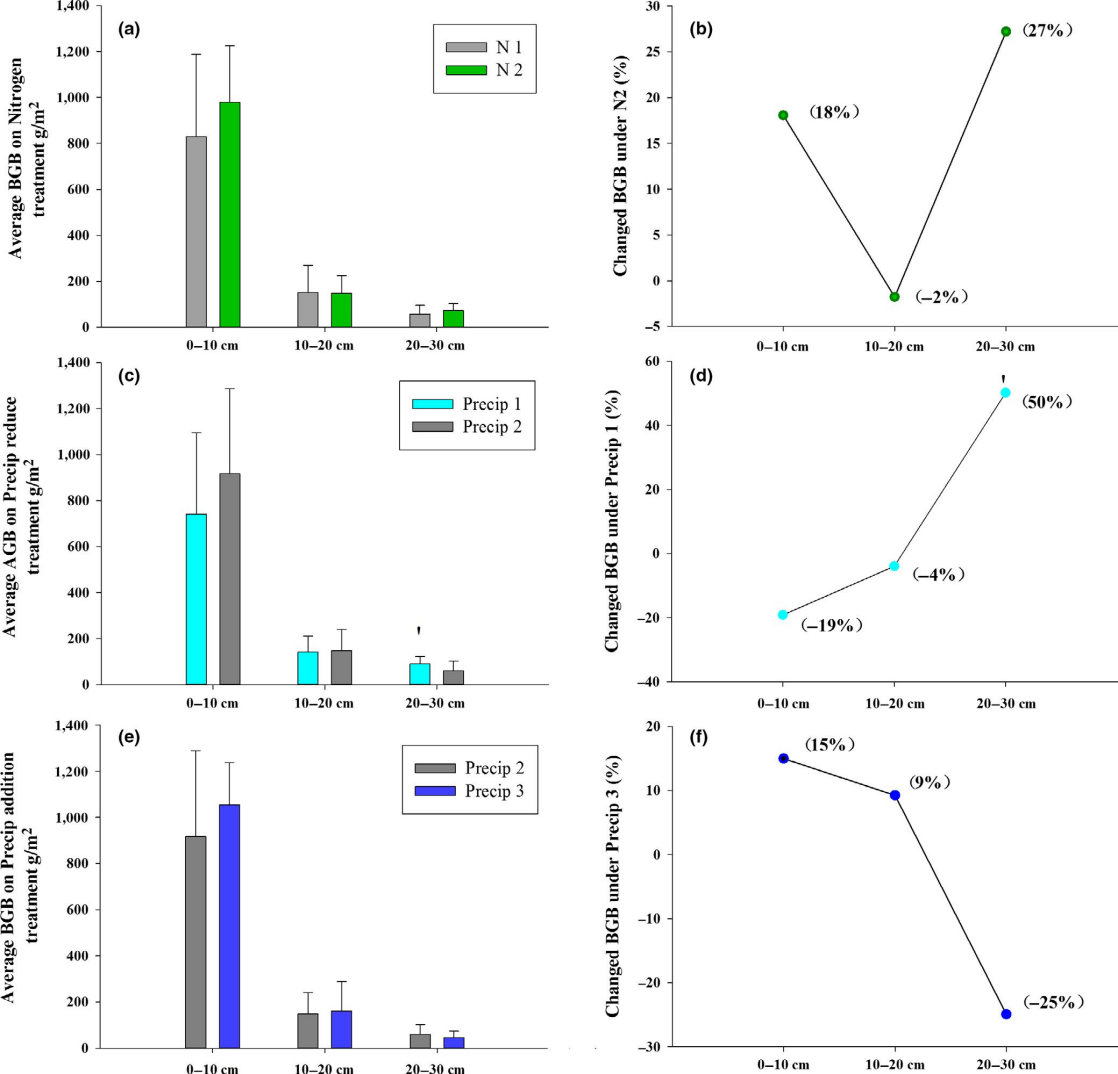
Effects of precipitation changes and N addition on belowground biomass (BGB) in 2016 (a, c, e) and changes (%) in belowground biomass (b, d, f) from 0–10 cm to 20–30 cm. N1 indicates ambient N, nitrogen addition (N2), 50% precipitation reduction (Precip 1), ambient precipitation (Precip 2), 50% precipitation addition (P3), ^′^ *p* < .1, error bars indicate the standard errors

To further explore whether belowground biomass in the different soil layers differs in their response to altered Precip, N addition, and their interaction, belowground biomass from 10, 20, and 30 cm deep soil was also determined. N addition and enhanced Precip had no effect on belowground biomass from three soil layers (Figure 3a,b,e,f). In contrast, belowground biomass from different soil layers differed in their responses to reduced Precip. Reduced Precip significantly increased biomass at 20–30 cm soil layers (*p* = .03), while it had little impacts on belowground biomass at 10 and 20 cm depth soil layers (Figure 3c,d; Table 3).

**Table 3:** Summary of univariate analysis of variance (UNIANOVA) of nitrogen addition and precipitation change on belowground biomass (BGB) in different soil depth in 2016

Difference source	*df*	Mean square	*F*‐value	*p*‐value
0–10 cm nitrogen (N)	1	168,390.09	1.61	.22
0−10 cm precipitation (Precip)	2	246,407.83	2.36	.12
0–10 cm N × Precip	2	16,130.92	0.15	.86
10–20 cm N	1	54.37	0.01	.95
10–20 cm Precip	2	996.06	0.09	.94
10–20 cm N × Precip	2	823.56	0.07	.93
20–30 cm N	1	1,805.97	1.43	.24
20–30 cm Precip	2	5,225.42	4.15	.03
20–30 cm N × Precip	2	897.05	0.71	.50

**Figure 4:**
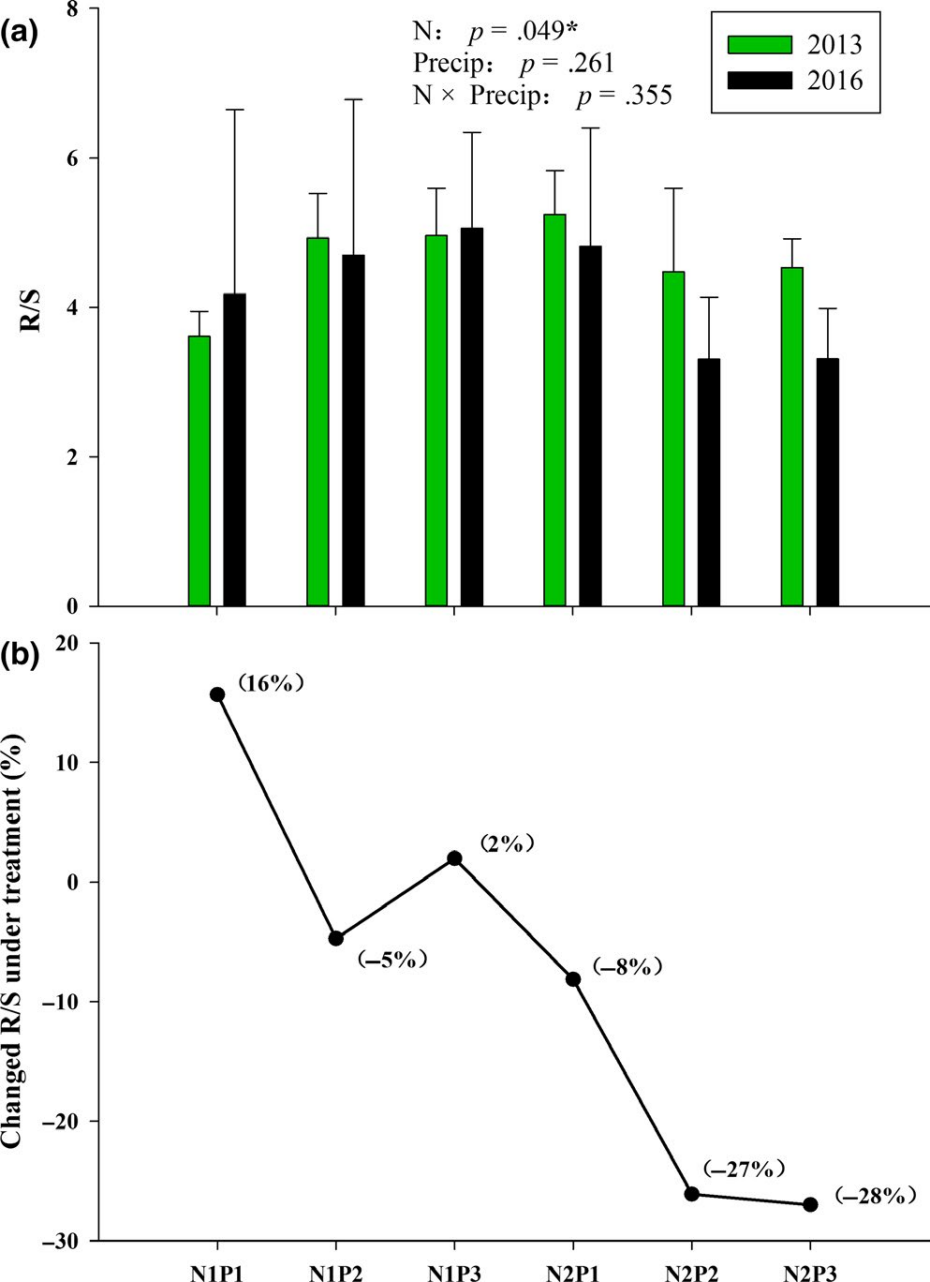
Effects of precipitation changes and N addition on changing of R/S ratios (a) and changes (%) in R/S (b). N_1_P_1_ indicates the 50% precipitation reduction treatment, ambient precipitation (N_1_P_2_), 50% precipitation addition treatment (N_1_P_3_), 50% precipitation reduction with nitrogen addition treatment (N_2_P_1_), the ambient precipitation without nitrogen addition (N_2_P_2_), 50% precipitation addition with nitrogen addition (N_2_P_3_), error bars indicate the standard errors

In this study, N addition significantly influenced the R/S ratio (*p* < .05; Table 4), reducing it by 8%, 26%, and 27% for N_2_P_1_, N_2_P_2_, and N_2_P_3_, respectively (Figure 4). Compared with the N addition, the effect of Precip on R/S was not significant (*p* = .26; Table 4).

**Table 4:** Summary of univariate analysis of variance (UNIANOVA) of nitrogen addition and precipitation change on changing of root: shoot ratio (R/S)

Difference source	*df*	Mean square	*F*‐value	*p*‐value
Nitrogen (N)	1	7,959.20	4.30	.049
Precipitation (P)	2	2,638.07	1.42	.26
N × P	2	2,008.54	1.08	.36

### 3.2 Effects of altered precipitation, N addition, and their interaction on aboveground–belowground biomass allocation

Consecutive N addition and altered Precip had differential effects on aboveground and belowground biomass allocation. N addition significantly reduced the ratio of root to shoot (R/S), leading to a marked increase in aboveground biomass allocation, while altered Precip and the interaction between altered Precip and N addition had little impacts on R/S (Figure 4; Table 4).

**Figure 5:**
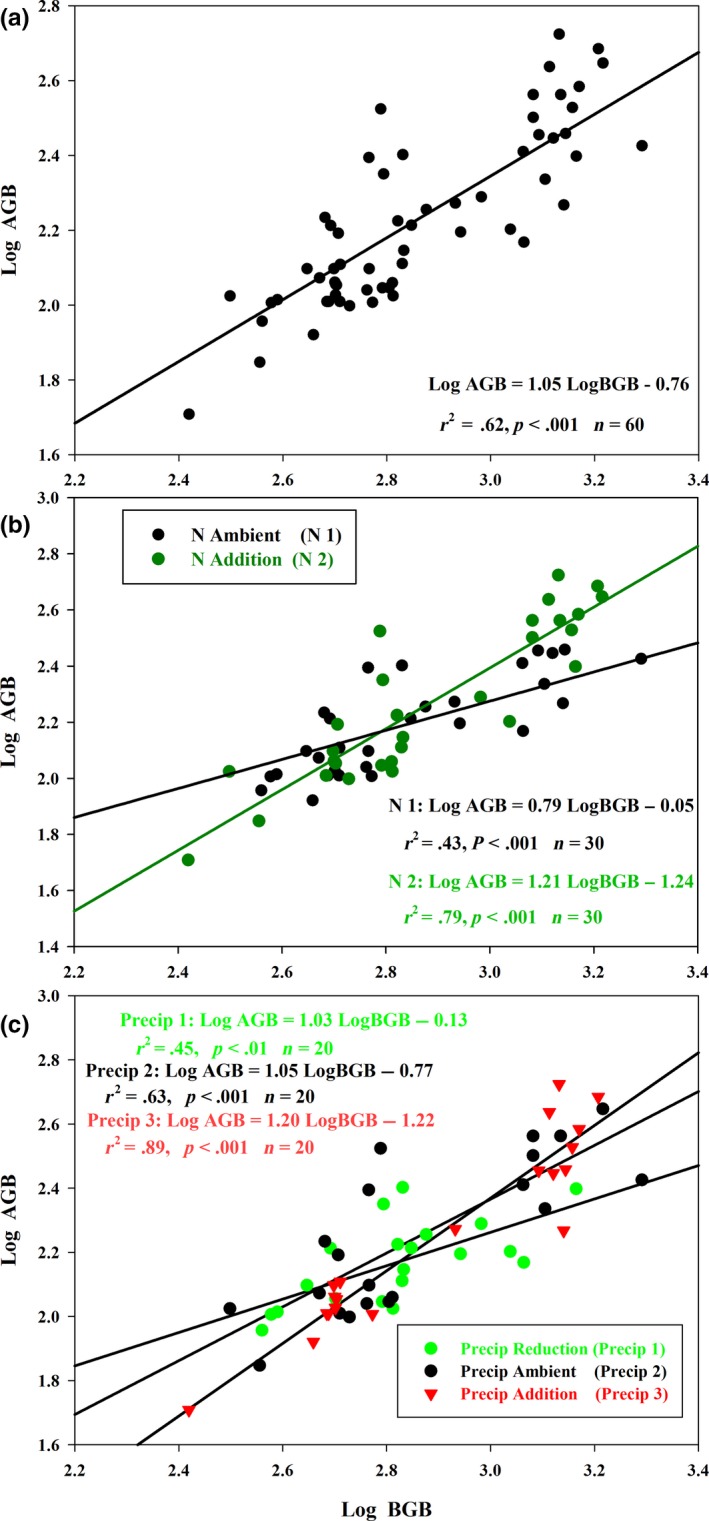
Relationships between aboveground biomass (AGB) and belowground biomass (BGB) in Tibetan Plateau. (a) The slope of the relationship between log AGB and log BGB for overall treatments was 1.06, with 95% confidence intervals of 0.89–1.23. (b) The black line denotes the allocation relationship for N ambient (N1), while the green line indicates the relationship for N addition (N2). The 95% confidence intervals of the slopes for N1 and N2 were 0.59–1.06 and 1.02–1.45, respectively. (c) The green indicates the allocation relationship for P reduction (Precip 1), and the black denotes the relationship for P ambient (Precip 2), while the red indicates the relationship for Precip addition (Precip 3). The 95% confidence intervals of the slopes for Precip 1, Precip 2, and Precip 3 were 0.72–1.48, 0.78–1.42, and 1.02–1.41, respectively

To further explore the relationships between N addition and altered precipitation, two allocation hypotheses were tested by testing plant biomass allocation under different N addition and precipitation condition. In natural alpine steppe, the isometric relationships between aboveground and belowground biomass allocation were observed (Figure 5a). In contrast, N addition and enhanced Precip significantly increased aboveground biomass allocation, leading to allometric allocations between aboveground and belowground biomass, while reduced Precip had no effect on the relationship between aboveground and belowground biomass allocation (Figure 5b,c).

## 4 DISCUSSIONS

Several manipulative experiments to simulate the changes in precipitation pattern and atmospheric N deposition have demonstrated that enhanced N input and enhanced precipitation increased aboveground plant biomass, while reduced precipitation led a marked decrease in aboveground plant biomass (Liu et al., 2018; Tian et al., 2016; Yang, Fang, Ma, Guo, & Mohammat, 2010). In the present study, we found that N addition significantly enhanced plant community aboveground biomass, while enhanced precipitation had no effect on aboveground plant biomass and reduced precipitation significantly decreased plant community aboveground biomass (Figure 1). These findings highlight that the alpine steppe was limited by soil N availability, but not water. This might be due to the fact that cold climates in cold alpine steppe depressed N availability and water vaporization (He et al., 2006, 2008).

Another important finding is that N addition had no effect on belowground biomass (Figure 2). In long‐term N‐limited grassland ecosystems, their native plant species have evolved mechanisms to cope with low N availability (Chapin, 1980). Exogenous N input into soil will lead to shift from being N limited to light limited (Bobbink et al., 2010; Hautier et al., 2009). Therefore, in contrast to the response of aboveground biomass to N addition, N addition hardly led to change in belowground biomass. In contrast, we found that enhanced precipitation significantly increased belowground biomass, while reduced precipitation markedly decreased belowground biomass (Figure 2). In the field, we observed that enhanced precipitation had no effect on community structure, while reduced precipitation favored the growth of deep‐root plant species, and suppressed shallow‐root plants, leading to loss of shallow‐root plant species (personal observation). However, we found that reduced precipitation significantly enhanced root biomass in deep soil layer, mainly due to that fact that reduced precipitation favored deep‐root plant growth to enhance the efficiency of water availability.

The isometric relationships between aboveground and belowground biomass were found in the natural alpine steppe of community levels (Figure 5). Some studies have also reported an isometric relationship between aboveground and belowground biomass, for example, Yang et al. (2009) and Enquist and Niklas (2002). These consistent results indicate that coexisting plant species have evolved some mechanisms to adapt their environments and to maintain a common growth (Falster et al., 2015). For example, in long‐term evolution, coexisting species occur inter‐ and intraspecific competition to acquire aboveground and belowground resources (Grace et al., 2016; Grime, 1974, 2006), leading to an isometric growth between shoot and root systems at the community level and allometric grow at the species level. Conversely, external resource input into soils and/or unsuitable conditions greatly affect the relationships between aboveground and belowground biomass allocations and carbon flux (Coomes, Holdaway, Kobe, Lines, & Allen, 2012; Jenkins & Pierce, 2017). In the present study, we found that N and water increment enhanced aboveground biomass allocation, leading to an allometric growth between aboveground and belowground plant biomass (Figure 5). This suggests that changes in environment will affect the balance between belowground and aboveground growth. More surprising, we found that decrease in the precipitation had little effect on aboveground and belowground biomass, still maintaining an isometric growth. However, more biomass allocation into deep soil was observed in the present alpine steppe ecosystem (Figure 3). More root biomass in deep soil suggests that reduced precipitation stimulates root proliferation in deep soil, thus enhancing the ability to acquire water resources (Lambers et al., 2008). Therefore, future studies on impacts of altered precipitation should take root biomass from different soil layers into account in the community level.

## 5 CONCLUSIONS

In summary, we demonstrated that altered precipitation and N addition led to changes in the relationship between aboveground and belowground biomass allocation. Specifically, in natural alpine steppe, aboveground and belowground biomass allocation conform to isometric hypothesis. In contrast, precipitation and N enrichment enhanced aboveground biomass allocation, leading to allometric allocation. Moreover, we demonstrated that reduced precipitation had little impacts on aboveground and belowground biomass allocation, while it enhanced more biomass into deep soil. Therefore, our finding provides a novel insight into N and precipitation‐induced changes in the aboveground and belowground biomass allocation in the alpine steppe.

## CONFLICT OF INTEREST

None declared.

## AUTHORS CONTRIBUTION

C.B.L., Z.Z., X.Q.N., L.C.Y., and Y.M.X. collected the data and participated in discussions. C.B.L. analyzed the data and wrote the manuscript with Z.Z., G.Y.Z., and Y.F.P.

## Data Availability

We had uploaded our data to the OSF. https://mfr.osf.io/render?url=https%253A%252F%252Fosf.io%252Fmd234%252Fdownload
