# Supplementary material for: Precipitation and nitrogen addition enhance biomass allocation to aboveground in an alpine steppe
Source: Ecol Evol. 2019 Oct 4;9(21):12193–201. doi: 10.1002/ece3.5706 (PMC6854108; doi:10.1002/ece3.5706)
Supplement: Supplementary file 1 [file ECE3-9-12193-s001.docx]

**Supporting information for manuscript entitled:** **Precipitation and nitrogen addition enhance biomass allocation to aboveground in an alpine steppe**

**Changbin Li^1,2,4^, Zhi Zheng^1,2^, Yunfeng Peng^3^, Xiuqing Nie^1,2,4^, Lucun Yang^1,2^, Yuanming Xiao^1,2,4^, Guoying Zhou^1,2*^**

^1^ Key Laboratory of Tibetan Medicine Research, Northwest Institute of Plateau Biology, Chinese Academy of Science, Xining 810008, China

^2^ Qinghai Key Laboratory of Qing-Tibet Biological Resources, Xining 810008, China

^3^ State Key Laboratory of Vegetation and Environmental Change, Institute of Botany, Chinese Academy of Sciences, Beijing 100093, China

^4^ University of Chinese Academy of Science, Beijing 100049, China

**^*^** Correspondence author: Guoying Zhou

Email address: [zhougy@nwipb.cas.cn](mailto:zhougy@nwipb.cas.cn)

Tel: +86-971-6159630

ORCID: <https://orcid.org/0000-0003-2485-6172>

Address: 23# Xinning Road, Xining, Qinghai, P. R. China 810008

**Supplement Table 1** The range and mean values of aboveground biomass (AGB), belowground biomass (BGB) and root: shoot ratio (R/S) for various treatments from 2013 to 2016. The experiments include six treatments (N1P1: ambient N addition with reduced precipitation 50%, N1P2: ambient N addition with ambient precipitation; N1P3: ambient N addition with enhanced precipitation 50%; N2P1: N addition with reduced precipitation; N2P2: N addition with ambient precipitation; N2P3: N addition with enhanced precipitation).

| **Treatments** | **AGB (g m^-2^)** | | **BGB (g m^-2^)** | | **R/S** | |
| --- | --- | --- | --- | --- | --- | --- |
|  | **Mean Values** | **Range** | **Mean Values** | **Range** | **Mean Values** | **Range** |
| **N1P1** | 144.917 | 116.709-  180.000 | 582.553 | 413.050-  752.057 | 3.895 | 3.612-4.178 |
| **N1P2** | 193.695 | 113.285-  232.021 | 822.725 | 556.035-  1089.416 | 4.812 | 4.695-4.929 |
| **N1P3** | 206.651 | 104.416-  259.883 | 872.193 | 506.360-  1238.027 | 4.987 | 4.918-5.055 |
| **N2P1** | 193.417 | 119.963-  250.571 | 748.078 | 536.750-  959.406 | 4.648 | 4.480-4.817 |
| **N2P2** | 271.608 | 109.173-  365.102 | 807.527 | 407.334-  1207.721 | 3.592 | 3.308-3.876 |
| **N2P3** | 308.169 | 101.333-  433.813 | 931.994 | 427.662-  1436.323 | 3.823 | 3.311-4.336 |


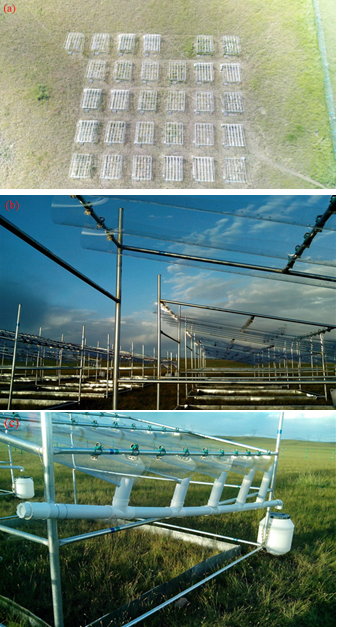


Supplement Figure 1 The manipulative experiments was conducted on the Sanjiaocheng Sheep Breeding Farm, in Qinghai province, China. (a) Aerial photographs of the experiments, (b) Six treatments in a row, (c) Precipitation reduced treatment. All of these photographs were taken by Changbin Li.
